# Supplementary material for: Multiparameter Flow Cytometric Analysis of the Conventional and Monocyte-Derived DC Compartment in the Murine Spleen
Source: Vaccines (Basel). 2024 Nov 19;12(11):1294. doi: 10.3390/vaccines12111294 (PMC11598974; doi:10.3390/vaccines12111294)
Supplement: Supplementary file 1 [file vaccines-12-01294-s001.zip › vaccines-3250774-supplementary-update.pdf]

Multiparameter flow cytometric analysis of the conventional and monocyte-derived DC compartment in the murine spleen

Ronald A. Backer, Hans Christian Probst, and Björn E. Clausen

Supplementary Information

Supplementary Materials

Fetal Bovine Serum (FBS):
Quickly thaw FBS at 37°C in a water bath. Once completely thawed, incubate for 60 min at 56°C in the water bath to destroy complement activity. Directly aliquot the warm FBS into 50 ml portions and store at -20°C. Avoid repeated freeze–thaw cycles. Use aseptic techniques during the whole procedure.
FACS buffer:
Phosphate-buffered saline solution (PBS) containing 3% FBS and 2 mM EDTA.
10× ACK (Ammonium-Chloride-Potassium) Lysing Buffer:
Dissolve 186.2 g EDTA in 800 ml H_20 and add 20 g of NaOH pellets. Adjust the pH to 8 and add MilliQ H_20 to 1000 ml. Store at room temperature and dilute the buffer to a 1-fold concentration prior to use.
Intracellular staining buffers:
Intracellular Langerin staining is performed using the reagents provided in the Cytofix/Cytoperm kit according to the manufacturer's instructions (BD Biosciences). For this, prepare the following buffers:

- A 1× working solution of Fixation/Permeabilization (Fix/Perm) buffer is provided.
- A 1× working solution of Permeabilization/Wash (Perm/Wash) buffer is used by mixing 1 part of the 10× concentrate with 9 parts of MilliQ water.

Alternatively, cells can be fixed with 4% Histofix and permeabilized using a Saponin buffer (FACS buffer containing 0.5% Saponin and 200 mM Glycine). Use the Saponin buffer to also dilute the anti-Langerin antibody and for the washing steps after intracellular staining.

Antibody Staining Mixes

Table S1. Cell surface antibody staining mix for flow cytometry

Excitation laser line	Fluorescence channel	Antibody	Dilution
Ultraviolet (355 nm)	BUV395	Anti-CD103	1/100
	BUV496	Anti-CD369 (anti-Dectin-1)	1/500
	BUV563	Anti-CD172a (anti-SIRPα)	1/400
	BUV615-P	Anti-FcεR1a	1/500
	BUV661	Anti-MerTK	1/500
	BUV737	Anti-F4/80	1/500
	BUV805	Anti-CD45pan	1/1000
Violet (405 nm)	BV421	Anti-CD371 (anti-Clec12A)	1/200
	BV480	Anti-Siglec-F	1/500
	BV570	Anti-Ly6C	1/500
	BV605	Anti-CD11b	1/500
	BV650	Anti-XCR1	1/500
	BV711	Anti-CD317	1/500
	BV750-P	Anti-Ly6G	1/500
	BV786	Anti-I-A/I-E (anti-MHC-II)	1/1000
Blue (488 nm)	BB515	Anti-CD24	1/400
	BB660-P	Anti-CD26	1/100
	BB700	Anti-CD64	1/500
	BB790-P	Anti-CX3CR1	1/200
Yellow-Green (561nm)	PE	Anti-ESAM	1/200
	PE-CF594	Anti-CD4	1/800
	PE-Cy7	Anti-CD8α	1/1000
	Dump (Lineage)	Anti-CD90.2	1/500
	Dump (Lineage)	Anti-CD19	1/500
	Dump (Lineage)	Anti-CD49b	1/500
	Dump (Lineage)	Anti-NK1.1	1/500
Red (637 nm)	APC-R700	Anti-CD11c	1/500
	FVS780	Live/Dead marker	1/1000

Table S2. Intracellular antibody staining mix for flow cytometry

Excitation Laser Line	Fluorescence Channel	Antibody	Dilution
Red (637 nm)	APC	Anti-CD207 (anti-Langerin)	1/200

BD FACSymphony™ Configuration

The 28-colour BD FACSymphony is equipped with five lasers:
355nm 100mW / 405nm 200mW / 488nm 200mW / 561nm 200mW / 637nm 140mW

Table S3. Instrument specifications and standard configuration

Excitation Laserline	Mirror	Filter	Flurochrome
355 UV	770 LP	810/40	BUV805
355 UV	690 LP	735/30	BUV737
355 UV	630 LP	670/25	BUV661
355 UV	595 LP	610/20	BUV615
355 UV	550 LP	580/20	BUV563
355 UV	450 LP	515/30	BUV496
355 UV		379/28	BUV395
405 Violet	750 LP	780/60	BV786
405 Violet	735 LP	750/30	BV750
405 Violet	685 LP	710/20	BV711
405 Violet	635 LP	677/20	BV650
405 Violet	595 LP	605/40	BV605
405 Violet	550 LP	586/15	BV570
405 Violet	505 LP	525/50	BV480
405 Violet	410 LP	431/28	BV421
405 Violet		405/10	SSC
488 Blue	750 LP	750/60	BB790-P
488 Blue	685 LP	710/50	BB700
488 Blue	635 LP	670/30	BB660-P
488 Blue	600 LP	610/20	BB630-P
488 Blue	505 LP	529/24	BB515
488 Blue		488/10	SSC
561 YG	750 LP	780/60	BYG790
561 YG	685 LP	710/50	PE-Cy5.5
561 YG	635 LP	670/30	BYG670
561 YG	600 LP	610/20	PE-CF594
561 YG	570 LP	586/15	BYG584
561 YG		560/10	SSC
633 Red	750 LP	780/60	APC-Cy7
633 Red	685 LP	730/45	APC-R700
633 Red	650 LP	670/30	APC
633 Red		630/20	SSC

LP = Long pass

Supplementary Figures

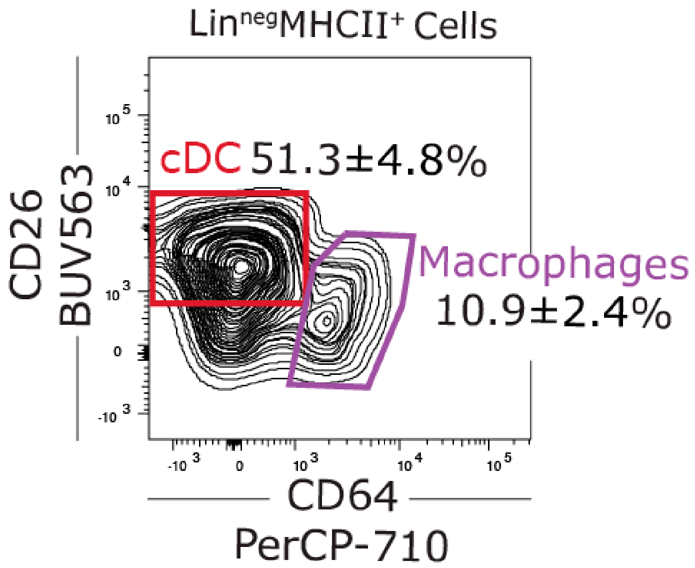

Figure S1: Gating for CD24^{+} cDC and CD64^{+} macrophages among Lin^{neg}MHC-II^{+} cells. Data acquisition was performed using a BD FACSymphony flow cytometer, and data analysis was performed using FlowJo software.

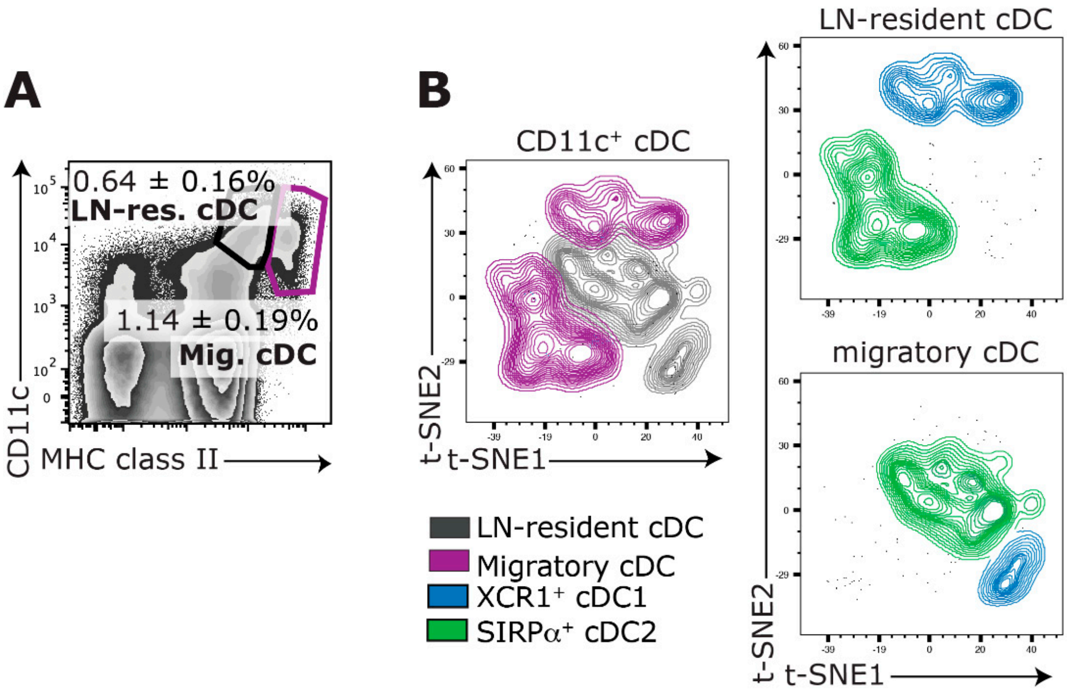

Figure S2: Phenotypic characterization of resident and migratory cDC1 and cDC2 in skin-draining LN. (A) t-SNE representation of peripheral LN cDC, with LN-resident cDC (*black*) and migratory cDC (*purple*). (B) Both populations can be further subdivided into XCR1^{+} cDC1 (*blue*) and SIRPα^{+} cDC2 (*green*) subsets. Data acquisition was performed using a BD FACSymphony flow cytometer, and data analysis was performed using FlowJo software.
